# Case Report: Hemorrhagic Vasculitis in a Patient With Multiple Sclerosis Receiving Fingolimod

**DOI:** 10.1002/ccr3.71449

**Published:** 2025-11-11

**Authors:** Farnaz Zeynali Esfahani, Maryam Payere, Bahar Karimikhoshnoudian, Mahdieh Baghaei, Mohammadali Nahayati

**Affiliations:** ^1^ Department of Neurology, Faculty of Medicine Birjand University of Medical Sciences Birjand Iran; ^2^ Department of Neurology, Faculty of Medicine Mashhad University of Medical Sciences Mashhad Iran; ^3^ Faculty of Medicine Islamic Azad University of Tehran Medical Sciences Tehran Iran; ^4^ Department of Radiology, Faculty of Medicine Mashhad University of Medical Sciences Mashhad Iran

**Keywords:** cerebral vasculitis, fingolimod, multiple sclerosis, opportunistic infection, varicella‐zoster virus

## Abstract

Fingolimod is an effective therapy for relapsing–remitting multiple sclerosis (RRMS) but predisposes patients to opportunistic infections, including varicella‐zoster virus (VZV). We report a case of a 27‐year‐old Iranian man with a history of MS, stable on fingolimod for two years, who presented with worsening paresthesia and headache. His condition acutely deteriorated, leading to a diagnosis of VZV‐associated cerebral vasculitis, intracerebral hemorrhage, and myelitis, confirmed via cerebrospinal fluid PCR and angiography. This case underscores that patients on fingolimod can develop severe, atypical neurological complications from VZV reactivation, even in the absence of cutaneous signs. A high index of suspicion and prompt diagnostic workup are crucial for early antiviral and immunomodulatory treatment to improve outcomes.

**Summary**

- This report describes a rare but catastrophic complication of fingolimod: VZV‐induced cerebral vasculitis leading to hemorrhagic stroke and concurrent myelitis.
- Clinicians must be aware that VZV reactivation can mimic an MS relapse and present without skin findings.
- A high index of suspicion and prompt diagnostic workup is essential, as outcomes depend on rapid initiation of antiviral and immunosuppressive treatment.

## 1 Introduction

Multiple sclerosis (MS) is a chronic inflammatory and autoimmune disorder of the central nervous system characterized by demyelination and axonal damage [1]. Fingolimod, a sphingosine 1‐phosphate (S1P) receptor modulator, is an effective disease‐modifying therapy for relapsing forms of MS. Its mechanism involves sequestering lymphocytes within lymphoid tissues, thereby reducing their migration to the central nervous system [2, 3].

While effective, fingolimod therapy is associated with an increased risk of opportunistic infections, notably varicella‐zoster virus (VZV) reactivation [4]. Comparative studies have shown a significantly higher rate of VZV DNA detection in patients receiving fingolimod compared to those on other therapies, underscoring the need for vigilant monitoring [4, 5]. In immunocompromised individuals, VZV can cause a small‐vessel vasculopathy, leading to severe neurological complications such as meningoencephalitis, ventriculitis, or myelitis, even in the absence of a characteristic cutaneous rash [6].

Given the potential for atypical and severe presentations, we report a rare case of intracerebral hemorrhage (ICH) and concurrent myelitis secondary to VZV vasculitis in a patient treated with fingolimod for relapsing–remitting MS. This case highlights a critical diagnostic consideration and underscores the importance of early recognition and management of this serious complication.

## 2 Case History

### 2.1 Initial Presentation and Medical History

A 27‐year‐old Iranian male with a four‐year history of relapsing–remitting multiple sclerosis (RRMS) was admitted to the hospital due to worsening paresthesia in all four limbs (left‐sided predominance) and mild headache. His disease‐modifying therapy (DMT) began with interferon β‐1a, which was switched to fingolimod (0.5 mg daily, Proprietary Name: Danelvin; Generic Name: Fingolimod; Dosage Form: Hard Gelatin Capsule; Nanoalvand Company) following a relapse. He had been clinically stable on fingolimod for two years with no reported adverse effects.

### 2.2 Hospital Course and Neurological Deterioration

**FIGURE 1:**
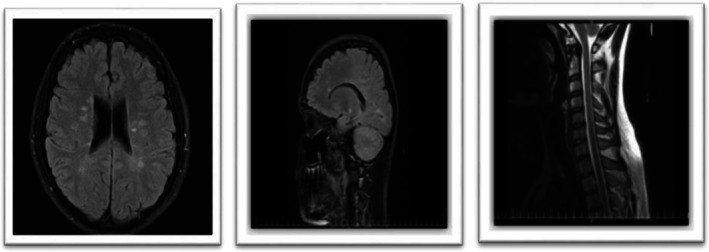
Baseline MRI at fingolimod initiation. Axial FLAIR brain MRI shows stable, non‐enhancing periventricular white matter lesions, consistent with the patient's known multiple sclerosis. And sagittal T2‐weighted cervical spine MRI at the same time demonstrates no evidence of myelitis or active spinal cord lesions.

**FIGURE 2:**
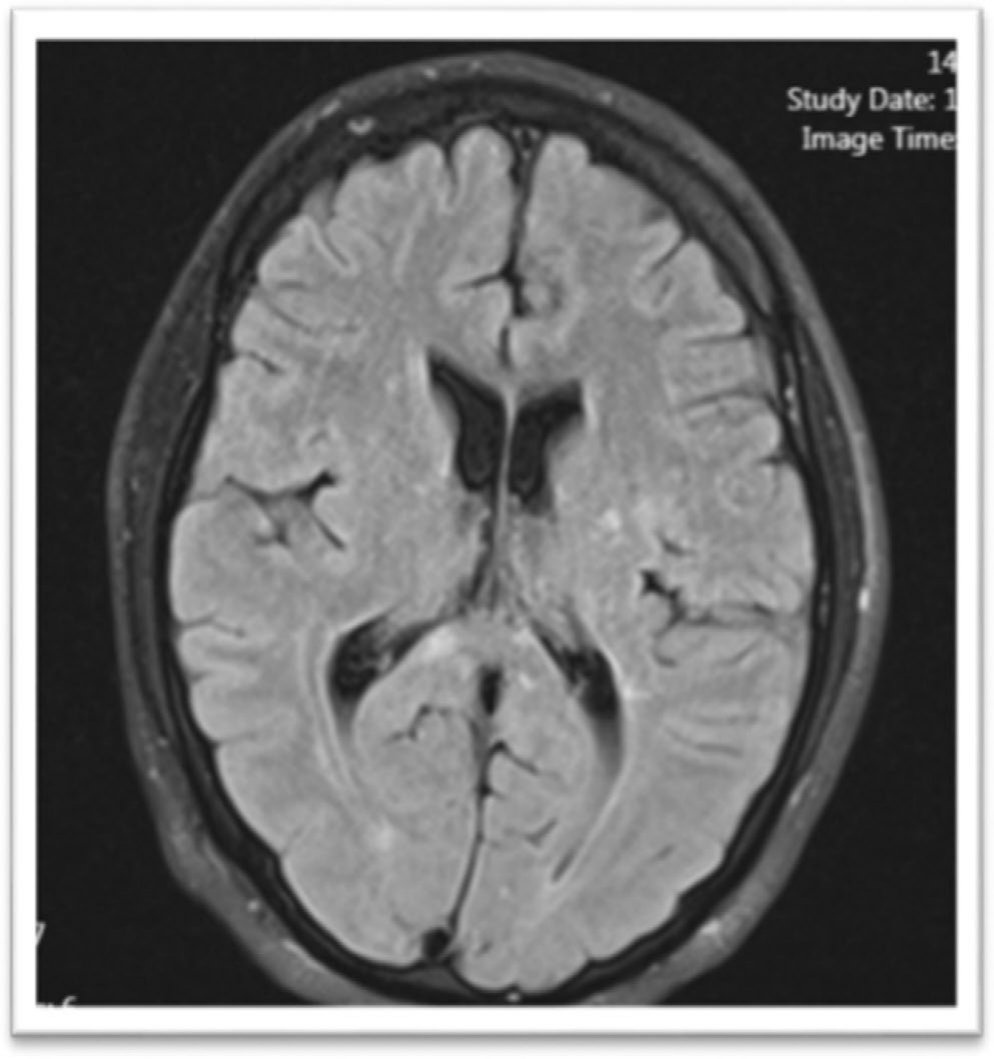
MRI on current admission. Axial FLAIR brain MRI reveals typical, stable MS plaques with no new enhancing lesions to explain the patient's acute neurological deterioration.

Upon admission, a new MS relapse was suspected. However, contrast‐enhanced MRI showed no interval changes compared to previous scans (Figures 1 and 2). Despite initiating intravenous corticosteroid therapy, the patient's condition worsened, developing into quadriparesis. Plasmapheresis was initiated. During the third session, he experienced acute neurological deterioration characterized by urinary incontinence, severe headache, fever, and worsening limb weakness.

### 2.3 Diagnostic Workup and Findings

**FIGURE 3:**
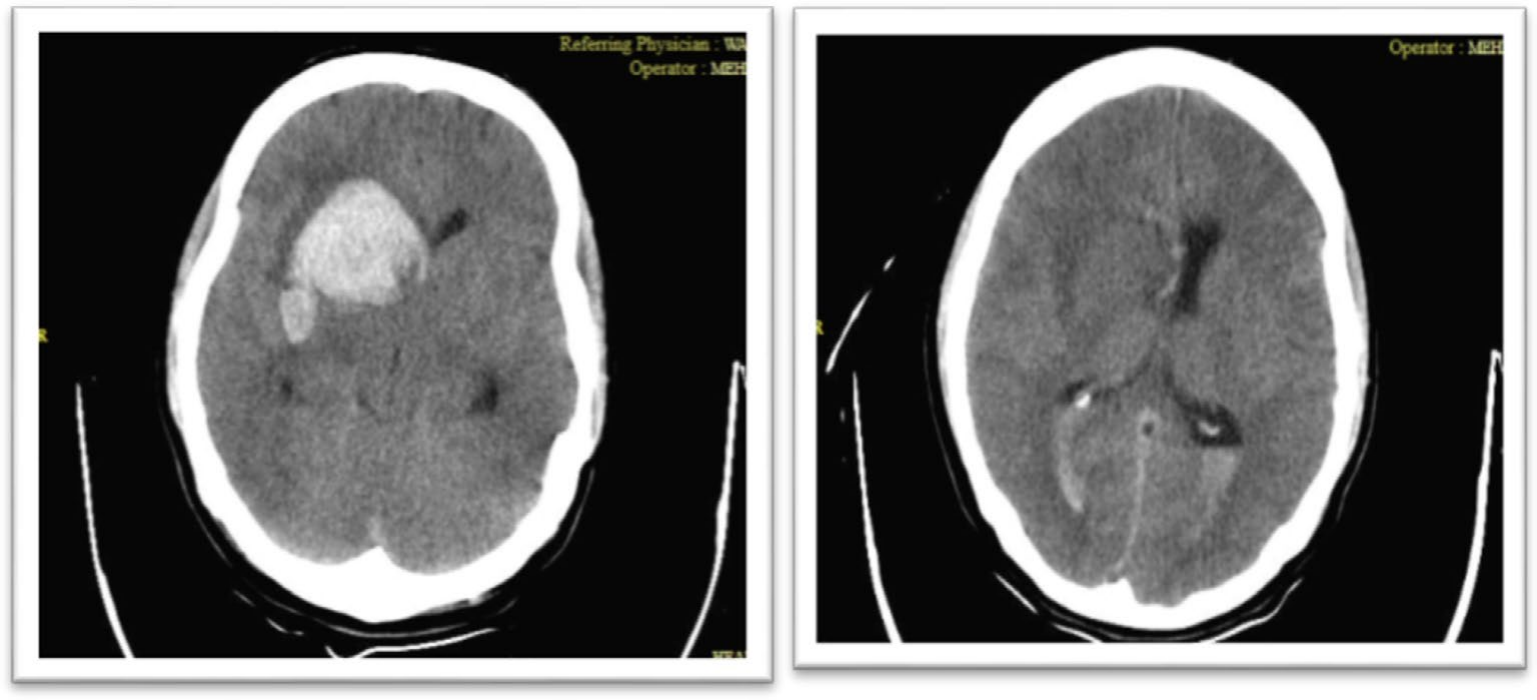
(Left) Axial image shows a large intracerebral hemorrhage in the right frontoparietal region extending to the basal ganglia, with significant surrounding edema causing mass effect and midline shift. (Right) A more superior slice demonstrates intraventricular hemorrhage within the lateral ventricles.

Given the fever and neck stiffness, lumbar puncture was performed. Subsequent computed tomography (CT) revealed an ICH in the right hemisphere (Figure 3). The patient became lethargic; his Glasgow Coma Scale (GCS) score dropped to 10, and he exhibited rightward gaze deviation, left‐sided facial and upper limb paresis, and a sensory level at T4. An emergent external ventricular drain (EVD) was placed to treat hydrocephalus.

**FIGURE 4:**
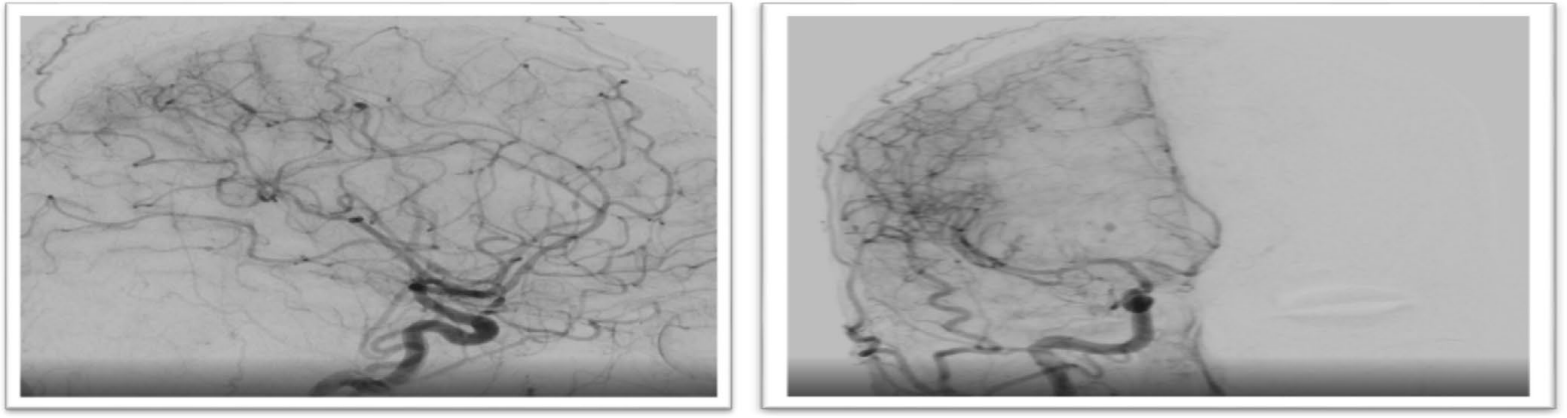
Anteroposterior view of the right internal carotid artery injection reveals multifocal areas of stenosis and dilatation, producing a “string‐of‐beads” appearance (arrows), diagnostic of cerebral vasculitis.

**FIGURE 5:**
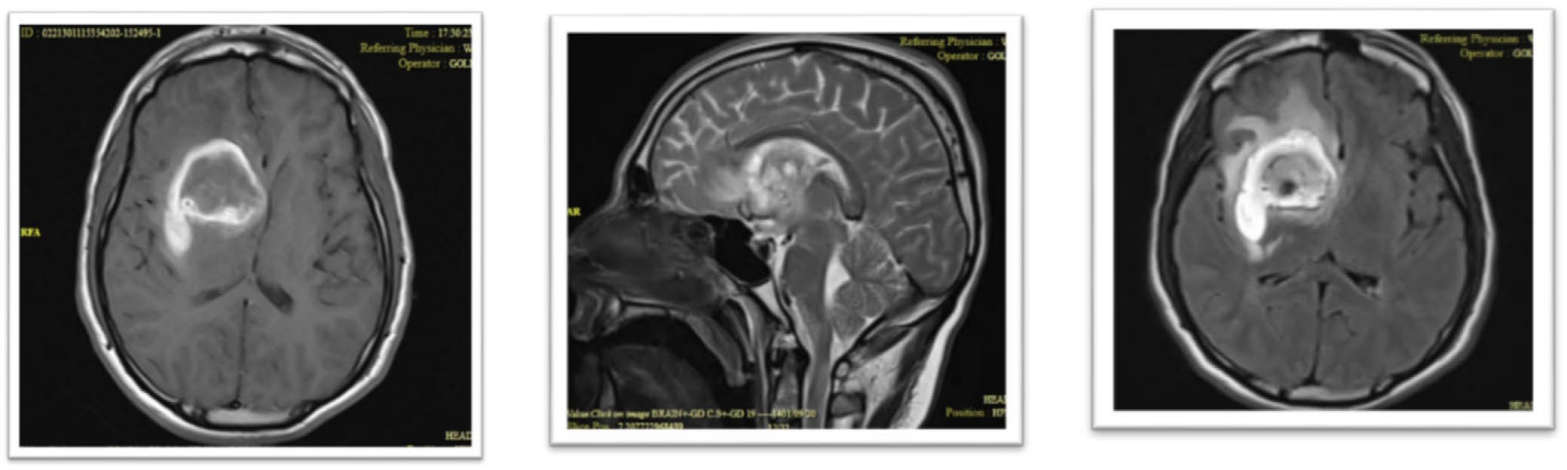
(Left) Axial T1‐weighted, (Middle) T2‐weighted, and (Right) FLAIR images demonstrate a heterogeneous signal focus in the right frontoparietal region consistent with acute hemorrhage, with extensive surrounding vasogenic edema.

**FIGURE 6:**
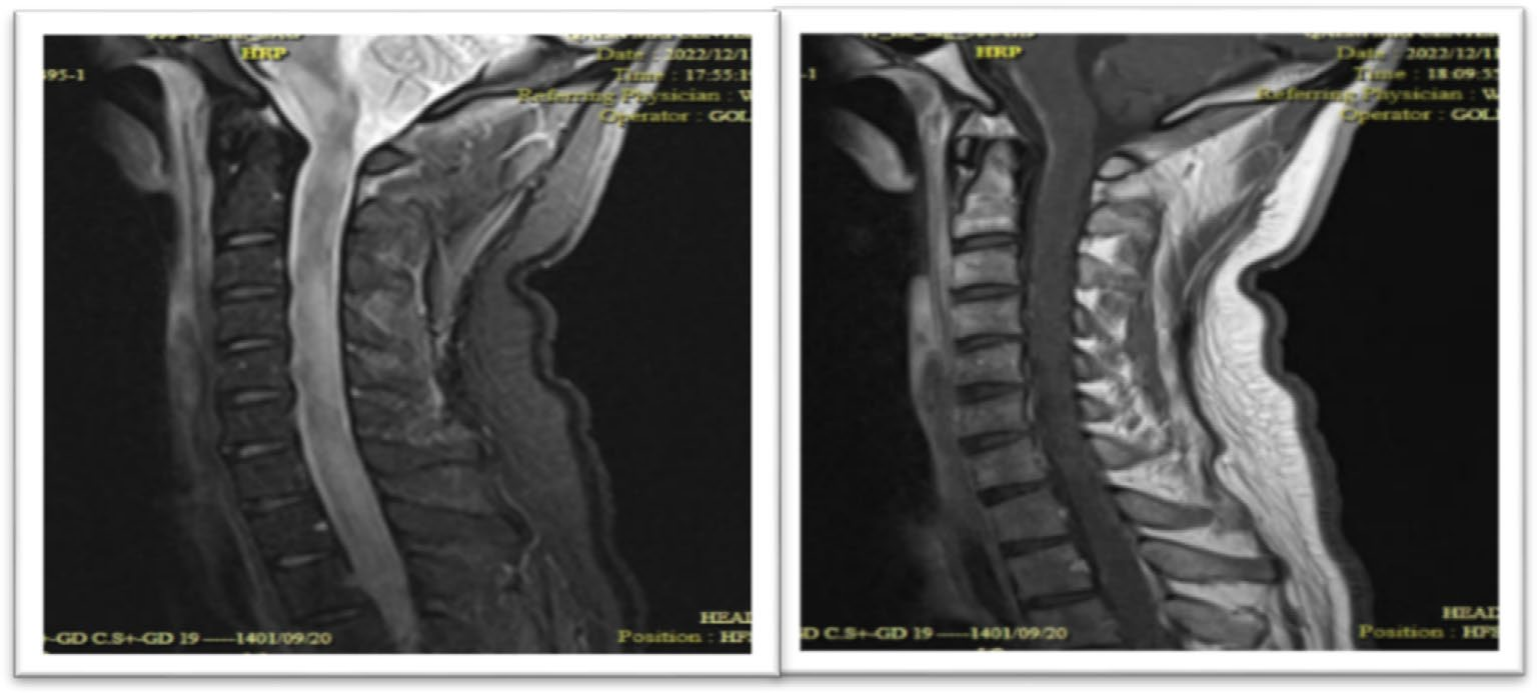
(Left) Sagittal T2‐weighted image shows a longitudinally extensive hyperintense lesion within the cervical spinal cord. (Right) Post‐contrast T1‐weighted image reveals prominent leptomeningeal enhancement, indicating active inflammation.

Digital subtraction angiography (DSA) demonstrated diffuse vessel beading, consistent with cerebral vasculitis (Figure 4). Postoperative MRI (Figures 5 and 6) confirmed the hemorrhage and surrounding edema.

Differential diagnoses, including infective endocarditis, were excluded via normal echocardiography. Cerebrospinal fluid (CSF) analysis was positive for oligoclonal bands but negative for common autoimmune and infectious panels. Crucially, VZV polymerase chain reaction (PCR) in the cerebrospinal fluid (CSF) returned positive, confirming VZV‐associated vasculitis. A brain biopsy ultimately demonstrated perivascular lymphocytic infiltration, corroborating the diagnosis.

**FIGURE 7:**
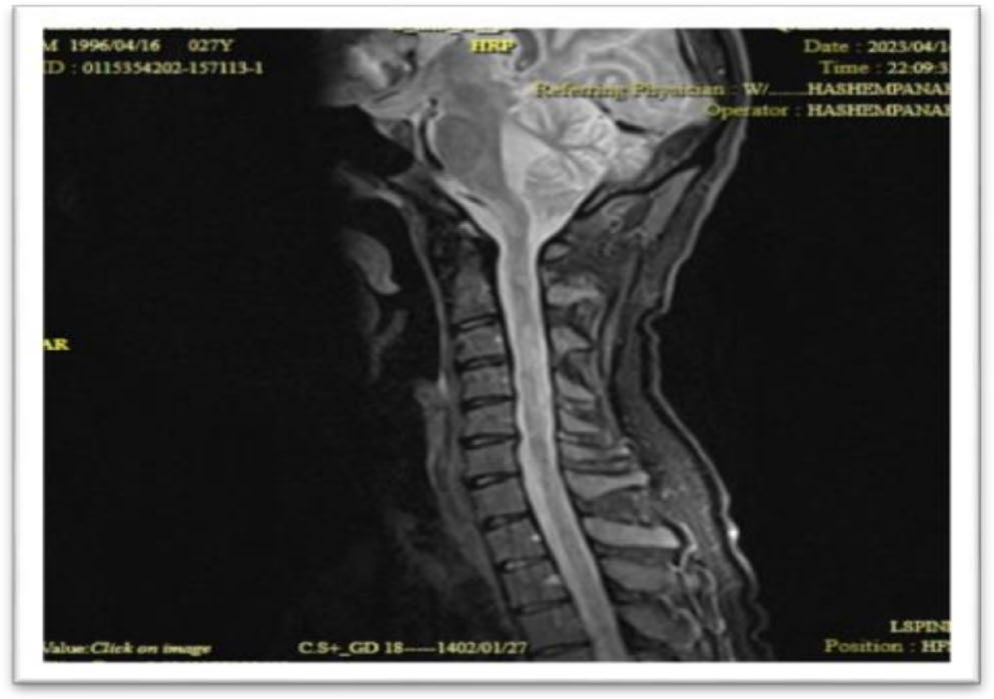
Sagittal T2‐weighted image shows significant regression of the previously noted hyperintense cord lesion, consistent with clinical improvement following antiviral and immunosuppressive therapy.

The patient received intravenous acyclovir (750 mg TID for 21 days) followed by cyclophosphamide. After two doses, his fever resolved, consciousness improved, and upper limb mobility partially returned. Due to concerns regarding fingolimod rebound and vasculitis progression, rituximab was initiated. Follow‐up imaging showed regression of cervical cord lesions (Figure 7).

## 3 Results

The patient was discharged after four months of hospitalization with significant neurological improvement. At discharge, he was afebrile, alert, able to follow commands, and exhibited partial motor recovery (upper limbs: 3/5 strength; lower limbs: 1/5 strength). Three months post‐discharge, he demonstrated further improvement, regaining partial lower limb movement (flexion). He was scheduled for subsequent rituximab infusions at six‐month intervals. By one‐year follow‐up, he had achieved independent ambulation with a cane, reflecting substantial functional recovery.

## 4 Discussion

Fingolimod is a highly effective disease‐modifying therapy for relapsing–remitting multiple sclerosis (RRMS). However, its immunosuppressive mechanism, which involves the sequestration of lymphocytes within lymphoid tissues, is a double‐edged sword, as it predisposes patients to specific opportunistic infections [2, 3]. This case vividly illustrates the severe neurological complications that can arise from such infections, specifically VZV reactivation.

### 4.1 Known Risks of Fingolimod and Opportunistic Infections

The association between fingolimod and an increased risk of Herpesviridae infections, particularly VZV, is well documented in the literature [4, 7]. Comparative studies have consistently shown a higher rate of VZV DNA detection and clinical reactivation in patients receiving fingolimod compared to those on other therapies [5]. Susceptibility to such atypical inflammatory responses may have a genetic component, as seen with polymorphisms in immune‐related genes like IRF5 and BLK in other neuroinflammatory disorders [8, 9]. While VZV can disseminate within the central nervous system (CNS) in immunocompetent individuals, immunosuppressed patients, like ours, are at a significantly higher risk for severe and atypical complications, including vasculopathy, even in the absence of the characteristic cutaneous zoster rash [6, 10]. This absence of a cutaneous herald can dangerously delay diagnosis, as the initial presentation may mimic an MS relapse.

### 4.2 Literature Review of VZV Vasculitis and Intracerebral Hemorrhage

VZV vasculitis typically presents with ischemic stroke; however, hemorrhagic transformation or primary ICH is a rare but documented and life‐threatening manifestation [11]. Our patient's radiological findings—diffuse vessel beading on angiography and a large frontoparietal hemorrhage—align with the established pathology of VZV‐induced vasculitis. Our findings are consistent with Wu et al. [12], who in a literature review identified six cases of VZV‐related cerebrovascular complications, underscoring the heterogeneity of presentations and the diagnostic challenge they pose. Furthermore, the concurrence of longitudinally extensive myelitis in our patient adds another layer of complexity, reflecting the virus's ability to cause multifocal CNS pathology. Jain et al. were among the first to highlight ICH as a presentation of VZV vasculitis, a finding that has been corroborated by subsequent case reports, including one by Muccilli et al. in a fingolimod‐treated patient [6, 10, 11].

### 4.3 Implications for Clinical Management and Future Vigilance

This case underscores several critical implications for clinical practice. First, a high index of suspicion is paramount. In any MS patient on fingolimod presenting with acute or stroke‐like neurological symptoms, VZV vasculitis must be considered a primary differential, even without a rash. Prompt diagnostic workup, including cerebrospinal fluid (CSF) PCR for VZV and vascular imaging (CTA, MRA, or DSA), is essential [13].

Second, early and aggressive treatment is crucial for improving outcomes. Immediate initiation of intravenous acyclovir (10–15 mg/kg) remains the cornerstone of therapy [14]. The role of concurrent corticosteroids to mitigate vascular inflammation is common practice, though their efficacy remains debated [12, 14]. For severe or refractory cases, as in our patient who only partially responded to acyclovir and cyclophosphamide, escalation to adjunctive immunomodulatory therapies like rituximab may be necessary to control the inflammatory vasculopathy [15].

Finally, this case highlights the need for meticulous risk stratification. When initiating or continuing fingolimod, particularly in VZV‐seropositive patients, physicians must carefully balance the drug's efficacy against the potential for severe opportunistic infections. Patient education regarding warning signs and a low threshold for investigating atypical neurological symptoms is an indispensable component of management.

## 5 Conclusion

This case provides three critical lessons for neurologists managing MS with modern immunotherapies. First, fingolimod‐associated VZV reactivation can manifest as a severe, multifocal CNS vasculopathy presenting with hemorrhage and myelitis, even in the absence of cutaneous zoster. Second, any atypical or sudden neurological deterioration in a patient on fingolimod should prompt immediate investigation for opportunistic infection, including CSF PCR for VZV and vascular imaging, to avoid a misdiagnosis of an MS relapse. Finally, successful management hinges on early, combined antiviral and immunomodulatory therapy, underscoring the need for vigilant monitoring and rapid intervention to mitigate the risk of catastrophic disability from these treatment‐related complications.

## Author Contributions

**Farnaz Zeynali Esfahani:** methodology. **Maryam Payere:** writing – original draft. **Bahar Karimikhoshnoudian:** writing – review and editing. **Mahdieh Baghaei:** writing – review and editing. **Mohammadali Nahayati:** conceptualization, supervision.

## Consent

Written informed consent was obtained from the patient to publish this report in accordance with the journal's patient consent policy.

## Conflicts of Interest

The authors declare no conflicts of interest.

## Data Availability

Data sharing is not applicable to this article as no new data were created or analyzed in this study.
